# Alternatively activated brain-infiltrating macrophages facilitate recovery from collagenase-induced intracerebral hemorrhage

**DOI:** 10.1186/s13041-016-0225-3

**Published:** 2016-04-19

**Authors:** Hyunjung Min, Yong Ho Jang, Ik-Hyun Cho, Seong-Woon Yu, Sung Joong Lee

**Affiliations:** Department of Neuroscience and Dental Research Institute, School of Dentistry, Seoul National University, 1 Gwanak-ro, Gwanak-gu, Seoul 08826 Republic of Korea; Department of Convergence Medical Science, College of Oriental Medicine, Kyung Hee University, Seoul, 02447 Korea; Department of Brain Science, Daegu Gyeongbuk Institute of Science and Technology, Daegu, 42988 Korea

**Keywords:** Immune response, Macrophages, Wound healing

## Abstract

**Background:**

Intracerebral hemorrhage (ICH) is one of the major causes of stroke. After onset of ICH, massive infiltration of macrophages is detected in the peri-hematoma regions. Still, the function of these macrophages in ICH has not been completely elucidated.

**Results:**

In a collagenase-induced ICH model, CX3CR1^+^ macrophages accumulated in the peri-hematoma region. Characterization of these macrophages revealed expression of alternatively activated (M2) macrophage markers. In the macrophage-depleted mice, ICH-induced brain lesion volume was larger and neurological deficits were more severe compared to those of control mice, indicating a protective role of these macrophages in ICH. In the ICH-injured brain, mannose receptor-expressing macrophages increased at a delayed time point after ICH, indicating M2 polarization of the brain-infiltrating macrophages in the brain microenvironment. To explore this possibility, bone marrow-derived macrophages (BMDM) were co-cultured with mouse brain glial cells and then tested for activation phenotype. Upon co-culture with glia, the number of mannose receptor-positive M2 macrophages was significantly increased. Furthermore, treatment with glia-conditioned media increased the number of BMDM of M2 phenotype.

**Conclusions:**

In this study, our data suggest that brain-infiltrating macrophages after ICH are polarized to the M2 phenotype by brain glial cells and thereby contribute to recovery from ICH injury.

## Background

Intracerebral hemorrhage (ICH) is a type of stroke characterized by blood leakage into the brain parenchyma due to blood vessel rupture within the brain. Although it accounts for only about 15 to 20 % of all strokes, ICH has a higher mortality and disability rate than other types of stroke. In ICH, the initial insult due to the mechanical force of the expanding hematoma and plasma proteins is usually followed by secondary damage, which involves inflammatory responses in the peri-hematoma region [1, 2]. The inflammatory responses accompany brain-resident astrocyte activation, compromise of the blood brain barrier (BBB) in the peri-hematoma region, and recruitment of peripheral immune cells such as neutrophils and macrophages to the injury site [3–7]. We have previously reported that ICH-induced neutrophil infiltration is partly responsible for secondary brain damage after ICH [8]. Also, we have shown that toll-like receptor 2 (TLR2)-mediated MMP9 activation in astrocytes is involved in the BBB compromise and subsequent neutrophil infiltration [8]. Other literature has documented that brain-infiltrated neutrophils exert neurotoxic effects by producing reactive oxygen species and pro-inflammatory mediators [9, 10]. Although a detrimental role of brain-infiltrating neutrophils after ICH has been well-documented, the role of brain-infiltrating macrophages has not been completely elucidated.

It is well known that macrophages display plasticity in their characteristics and can change phenotype and functions depending on the microenvironment [11, 12]. Typically, stimulation with lipopolysaccharide (LPS) and interferon-γ activates macrophages to express pro-inflammatory mediators such as TNF-α and iNOS, referred to as “classically activated” M1 macrophages [13]. Conversely, interleukin (IL)-4 and IL-10 stimulation induce “alternatively activated” M2 macrophages that promote tissue healing and angiogenesis [14]. These two types of macrophage activations are detected in various neurological injury models. In spinal cord injury, prolonged activation of M1 macrophages due to TNF-α and iron impedes recovery after injury [15], while TLR2-dependent M2 polarization of microglia protects against spinal cord injury [16]. In a murine ischemic stroke model, macrophages assume the M2 phenotype in early stages of ischemic stroke but gradually transform into the M1 phenotype in the peri-infarct region, which exacerbates neuronal death [17]. The phenotype of macrophages in hemorrhagic stroke is not yet completely understood. In this study, we used a mouse model of collagenase-induced ICH to explore the function and phenotype of brain-infiltrating macrophages after hemorrhagic stroke.

## Methods

### Animals

*Cx3cr1*^*+/GFP*^ mice with GFP knocked into the gene encoding CX3CR1 were from Jackson Laboratory. Seven- to 11-week-old C57BL/6 mice were purchased from Daehan Bio Link (Eumsung, Korea). All mice were housed at 23 ± 2 °C with a 12 h light–dark cycle and fed food and water *ad libitum*. All surgical and experimental procedures were reviewed and approved by the Institutional Animal Care and Use Committee at Seoul National University.

### ICH model

The procedure for inducing ICH through collagenase injection in mice was adapted from a protocol previously described [10]. Briefly, WT and *Cx3cr1*^*+/GFP*^ mice (8–12-week-old males, 22–25 g) were anesthetized using Avertin (250 mg/kg body weight, i.p.) and placed on a stereotaxic apparatus (myNeuroLab, St. Louis*,* MO, USA). Animals were injected with PBS or collagenase VII-S (0.075 U in 0.5 μl PBS; Sigma, St. Louis, MO, USA) at a rate of 0.2 μl/min into the right caudate putamen (stereotaxic coordinates in millimeters with reference to bregma: AP, −0.9; ML, +2.8; DV, −4.0) using a 26-G needle. After 5 min, the needle was removed in three intermediate steps for 3 min to minimize backflow. The incision was disinfected with povidone iodide solution and sutured, and the animals were kept on a warm pad during recovery.

### Injury volume analysis

To prepare brain tissue sections, animals were deeply anesthetized with urethane and perfused intra-cardially with saline followed by cold 4 % paraformaldehyde (PFA) in a 0.1 M phosphate buffer. The brains were removed, post-fixed overnight in the same fixative at 4 °C, rinsed twice with PBS, and placed serially in 10, 20, and 30 % sucrose in PBS for 72 h at 4 °C. The brains were then quickly frozen and cut into serial coronal sections (50 μm thickness) in a cryostat (CM3050S, Leica, Germany). Sections were collected as free-floating sections in cold PBS and then used for histological analysis. Five coronal brain slices from different levels of the injured hemorrhagic area were selected from each mouse brain and used for cresyl violet staining. The injury area was quantified using Image J software (National Institutes of Health, Bethesda, MD, USA), and the injury volume was calculated in cubic millimeters (mm^3^) by multiplying section thickness by measured injury area.

### Behavioral test

The adhesive removal test (ART) was performed as previously described [18]. Mice were habituated for at least 30 min prior to applying the sticky tape on the sole of the left fore paw. The removal time was defined as the period from the mouse’s initial reaction to the presence of the adhesive tape strips to the time it detached the tape using its mouth or forelimb. The duration of the procedure to test one mouse was 300 s maximum. ART was performed at 1 and 3 days after ICH or sham operation. Neurological function was measured by scoring focal deficits on a 28-point neurological scoring system [19]. Habituated mice were scored on the following measures: (1) Body symmetry, (2) Gait, (3) Climbing, (4) Circling behavior, (5) Front limb symmetry, (6) Compulsory circling, and (7) Whisker response. One and 3 days after ICH, scoring was performed on each subject on a scale from 0 to 4, and the scores were averaged.

### Peripheral macrophage/monocyte depletion

Peripheral monocytes/macrophages were systemically depleted using a clodronate liposome suspension (ClodronateLiposome.com, Amsterdam, Netherlands). Mice were injected intra-peritoneally with 1 mg of clodronate liposome (Cl_2_MDP) or saline 1 day before ICH surgery. One day after ICH, an additional injection was administered in order to increase the depletion rate. To measure depletion efficiency, the brains and blood samples were collected and monocyte/macrophage populations were evaluated by flow cytometry or anti-CD68 immunofluorescence staining.

### Immunohistochemistry

Immunostaining was carried out using previously established protocols [20]. The sections were incubated in a blocking solution (5 % normal donkey serum, 2 % BSA, and 0.1 % Triton X-100) for 1 h at room temperature (RT). The sections were then incubated overnight at 4 °C with goat anti-Arginase-1 (1:50; Santa Cruz Biotechnology, CA, USA) and rat anti-CD68 (1:100; AbD Serotec, Oxford, UK). The sections were incubated for 1 h at RT with Cy3-conjugated secondary antibodies (1:200; Jackson ImmunoResearch, West Grove, PA, USA) and then mounted on gelatin-coated slides and cover-slipped with VectaShield medium (Vector Labs, Burlingame, CA, USA). All images were acquired using confocal laser scanning microscopy (LSM700; Carl Zeiss, Germany).

### Quantitative real-time RT-PCR

The cDNA was synthesized using total RNA from mouse brain tissue or in vitro cultured cells. The reverse transcription mixture consisted of 1–3 μg of total RNA, oligo-dT, M-MLV, RNAse inhibitor, DTT, and RT buffer and was synthesized at 37 °C for 1 h. Real-time RT-PCR was performed using SYBR Green PCR Master Mix (ABI, Waltham, MA, USA) as previously described [20]. Reactions were performed in duplicate in a total volume of 12 μl containing 10 pM primer, 5 μl cDNA, and 6 μl SYBR Green PCR Master Mix (ABI). The mRNA level of each target gene was normalized to that of GAPDH mRNA. Fold-induction was calculated using the 2^-∆∆CT^ method, as previously described [21]. All real-time RT-PCR experiments were performed at least three times and are presented as mean ± SEM unless otherwise noted. The following sequences of primers were used for real-time RT-PCR: Arginase-1 forward: 5′-GGG CTC TGA TGA GAA GGA GA-3′; Arginase-1 reverse: 5′-GTA GAT GCC ACG CTG GTA CA-3′; Ym1 forward: 5′-GAA GGA GCC ACT GAG GTC TG-3′; Ym1 reverse: 5′-CAC GGC ACC TCC TAA ATT GT-3′; iNOS forward: 5′-GGC AAA CCC AAG GTC TAC GTT-3′; iNOS reverse: 5′-TCG CTC AAG TTC AGC TTG GT-3′; TNF-α forward: 5′-AGC AAA CCA CCA AGT GGA GGA-3′; TNF-α reverse: 5′-GCT GGC ACC ACT AGT TGG TTG T-3′; CD16 forward: 5′- TTT GGA CAC CCA GAT GTT TCA G-3′; CD16 reverse: 5′ GTC TTC CTT GAG CAC CTG GAT C-3′; CD86 forward: 5′- TTG TGT GTG TTC TGG AAA CGG AG-3′ and CD86 reverse: 5′- AAC TTA GAG GCT GTG TTG CTG GG-3′.

### Primary mouse brain glial cell culture

Primary mouse brain glial cultures were prepared as previously described [22]. Briefly, brains were prepared from postnatal day 1–3 wild-type mice. After removing the meninges from the cerebral hemisphere, tissue was dissociated into a single-cell suspension through gentle repetitive pipetting. Cells were cultured in Dulbecco’s Modified Eagle’s medium (DMEM) and supplemented with 10 mM HEPES, 10 % FBS, 2 mM L-glutamine, 1X non-essential amino acids (NEAA), and 1X antibiotic/antimycotic in 75 cm^2^ flasks at 37 °C in a 5 % CO_2 _ incubator. The medium was changed every 5 days.

### BMDM culture

The protocols for BMDM culture and cryopreservation were adapted from a previous study [23]. Briefly, femurs and tibias were obtained from 8 to 12-week-old C57BL/6 or *Cx3cr1*^*+/GFP*^ mice. The bones were flushed with a syringe filled with DMEM to extrude bone marrow into a 50 ml sterile tube. The cells were gently homogenized using a plastic pipette and aliquoted in freezing media containing 90 % FBS and 10 % DMSO. The aliquots were maintained at −80 °C for 24 h and then transferred to a liquid nitrogen tank. Bone marrow cells were thawed, washed with warm-DMEM, and then cultured in DMEM supplemented with 10 mM HEPES, 10 % FBS, 2 mM L-glutamine, 1X NEAA, 1X antibiotic/antimycotic, and 30 ng/ml recombinant human macrophage colony stimulating factor (rhM-CSF; Peprotech, Rocky Hill, NJ, USA) at 37 °C in a 5 % CO_2_ atmosphere for 5 days. For mRNA quantification or flow cytometry analysis, BMDMs were seeded in a 6-well plate (2.5–5 × 10^5^/well) with growth media supplemented with 10 ng/ml rhM-CSF for 2 days.

### Co-culture experiment

While the BMDM were removed from the plate with a scraper, the primary mixed glial cells were simultaneously detached using trypsin. Cells were counted using a hemocytometer (2.5–5 × 10^5^ cells) and then added to a 60 mm culture dish at a 1:1 ratio in BMDM growth medium-containing 10 ng/ml rhM-CSF. Co-cultures were incubated at 37 °C in a CO_2_ incubator.

### Glial cell-conditioned media treatment

Two weeks after glial culture, conditioned media were collected. To prevent cell contamination, conditioned media were centrifuged for a short time and then stored in a −80 °C deep freezer. A 1:1 mixture of glia-conditioned media and BMDM growth media was used for treatment.

### Flow cytometry

The mice were deeply anesthetized with urethane and intra-cardially perfused with ice-cold saline. Ipsilateral hemispheres were trimmed on the anterior and posterior sides from the needle hole, and then residues of brain hemispheres were homogenized mechanically to a single cell-suspension. In addition, blood samples were collected by retrobulbar puncture before mice were perfused with saline and red blood cells were removed by RBC lysis buffer (150 mM NH_4_Cl, 10 mM KHCO_3_, 100 μM EDTA). For the analysis of cultured cells, co-cultures or BMDMs were removed from the plates with cold PBS and re-suspended using a pipette. Cells were washed with ice-cold 2 % FBS in PBS and incubated with Fc Blocker^TM^ (BD Bioscience, San Jose, CA) for 10 min at 4 °C prior to staining with CD11b-FITC, CD45-PE, CD206-APC, F4/80-PE/Cy7 (Biolegend Inc., San Diego, CA). BD FACSCalibur flow cytometer (BD Bioscience) was used to measure the microglia as CD11b^+^/CD45^low^, peripheral macrophage as CD11b^+^/CD45^high^, and BMDM as CD45^+^/GFP^+^ or CD11b^+^/CD45^+^. Data were acquired and analyzed with BD CellQuest^TM^system (BD Biosciences).

### Statistical analysis

Statistically significant differences between two groups were determined using two-tailed Student’s *t*-test. Differences among multiple groups were identified using one-way ANOVA followed by Bonferroni correction. All data are presented as mean ± SEM, and differences were considered significant when the *p-*value was less than 0.05.

## Results

### ICH injury induces macrophage infiltration into injured brain parenchyma

**Fig. 1:**
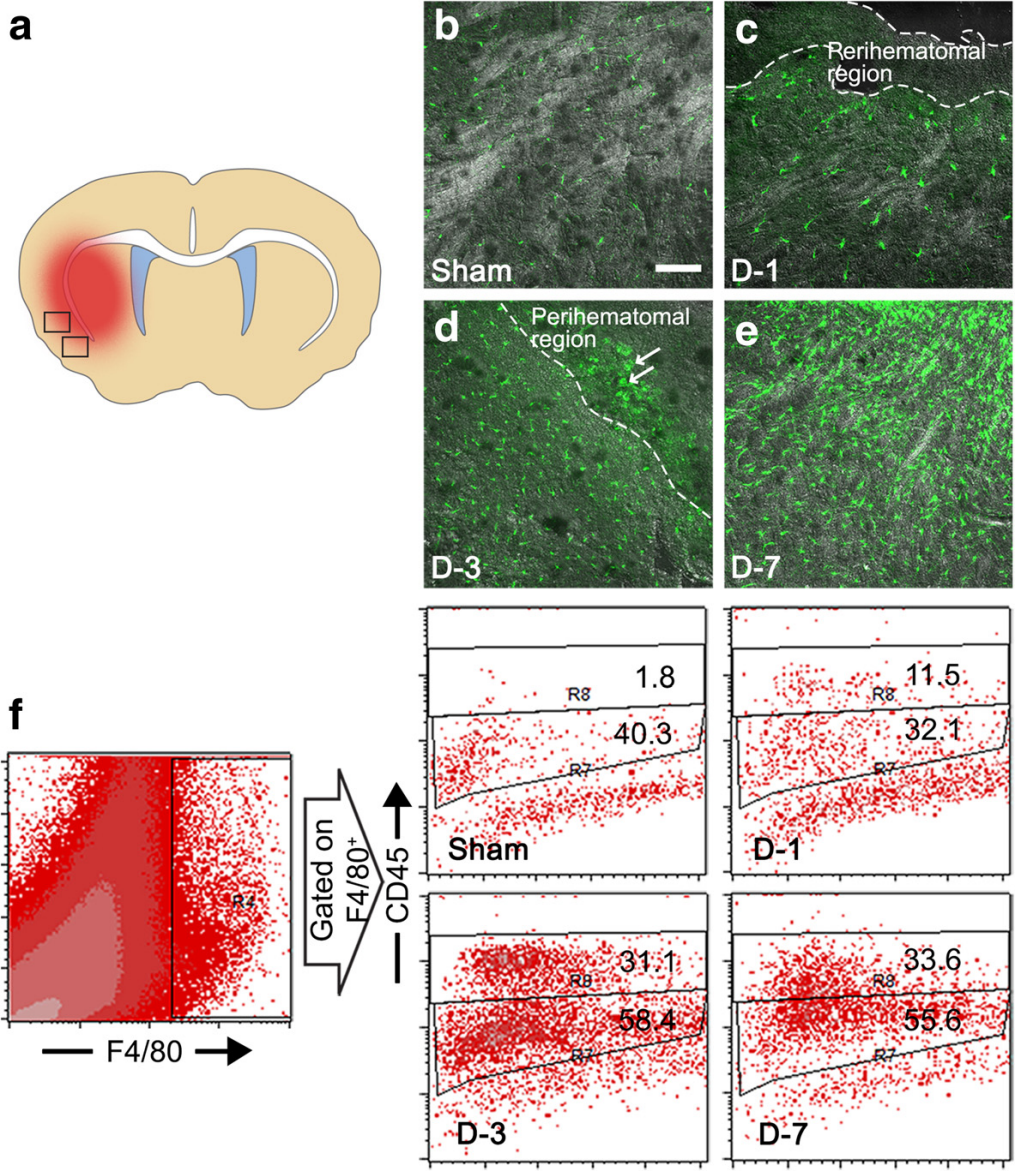
CX3CR1^+^ cells increase in the peri-hematoma region after ICH. **a** CX3CR1^+^ cells were detected in peri-hematoma regions of hemorrhagic brain sections. **b–e** Representative images on brain sections were obtained from *Cx3cr1* ^*+/GFP*^ mice at 1, 3, and 7 days after ICH or sham operation. Three days after ICH, amoeboid cells were detected in the peri-hematoma region (*arrows*) (Scale bar, 100 μm). **f** Myeloid cells (F4/80^+^) in the lesion were analyzed and further divided into CD45-low microglia and CD45-high macrophage populations according to the expression level of CD45 according to flow cytometry. The flow cytometry data are representative of three independent experiments

To detect macrophages infiltrating brain parenchyma after ICH injury, we utilized *Cx3cr1*^*+/GFP*^ mice in which CX3CR1-expressing macrophages and microglia can be monitored using GFP fluorescence. Following ICH, the cells with GFP fluorescence increased as soon as 1 day post-injury (dpi) in the surrounding area of hematoma, indicating upregulation of CX3CR1 in microglia/macrophages in this area (Fig. 1b and c). Three days after ICH, CX3CR1^+^ cells greatly increased in the injured brain area. In particular, round CX3CR1^+^ cells, morphologically presumed to be brain-infiltrating activated macrophages, accumulated in the peri-hematoma region, which was further increased on 7 dpi (Fig. 1d and e). To differentiate macrophages from microglia in this CX3CR1^+^ population, we analyzed the F4/80-positive monocytic cell population in the injured brain using flow cytometry based on the expression level of CD45 (Fig. 1f) [24]. The percentage of CD45^high^/F4/80^+^ leukocytes representing the macrophage population increased to 11.5 % on 1 dpi and 31 % on 3 dpi from 1.8 % (sham). In the same time frame, the number of CD45^low^/F4/80^+^ cells representing the microglia population was not as significantly altered; it increased only to 58 % on 3 dpi from 40 % (sham). These data demonstrate that the increase in CX3CR1^+^ cells after ICH is mainly due to macrophage infiltration rather than microglia proliferation.

### Brain-infiltrating macrophages following ICH show M2 phenotype

**Fig. 2:**
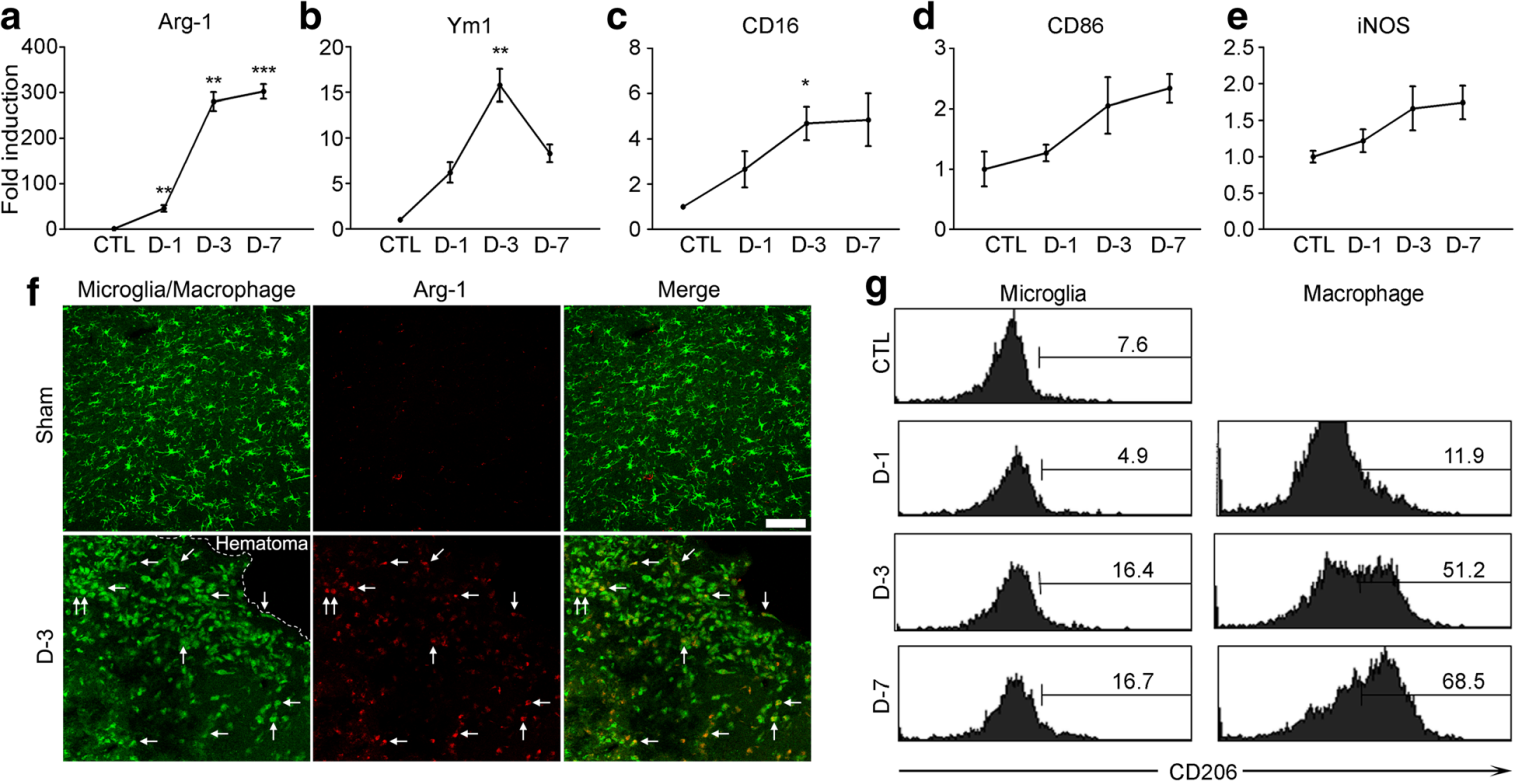
M2 macrophages are increased in the ipsilateral brain after ICH. **a-e** mRNA expressions of M2 markers (Arg-1 and Ym1) or M1 markers (CD16, CD86 and iNOS) in the ICH mice brains were measured using real-time RT-PCR. Total RNA was isolated from ipsilateral hemorrhagic tissue and used for quantitative real-time RT-PCR (*n* = 3 per group, **p < 0.01, ***p < 0.001). **f** Representative images of Arg-1 immunofluorescence (*red*) in the *CX3CR1* ^*+/GFP*^ mice brain sections obtained from ICH or sham-control mice. Arg-1 immunofluorescence merged with myeloid cell-specific GFP signals is denoted (*arrows*) (Scale bar, 100 μm). **g** Dissociated cells from the injured tissue were obtained 1, 3, and 7 days after ICH injury and were used for flow cytometry with anti-CD11b-FITC, −CD45-PE, and -CD206-APC antibodies. Representative flow cytometry histograms show CD206^+^ cells gated on CD11b^+^/CD45^+^ populations. The flow cytometry data are representative of three independent experiments

To characterize the phenotype of the brain-infiltrating macrophages, we analyzed expression of typical M1 (CD16, CD86, and iNOS) and M2 (Arginase-1 and Ym1) markers in the injured brain. Upon initiation of ICH, the mRNA expression of Arginase-1 increased more than 300-fold in the injured brain at 7 dpi. Likewise, Ym1, another M2 marker, expression was increased 15-fold at 3 dpi (Fig. 2a and b). For M1 markers, only CD16 expression was increased 4.7-fold at 3 dpi (Fig. 2c), whereas other M1 markers such as CD86 and iNOS expressions were not significantly altered (Fig. 2d and e). Arginase-1 expression was mainly detected in CX3CR1^+^ macrophages/microglia in the peri-hematoma region (Fig. 2f, arrows), suggesting that macrophages/microglia in the injured brain are polarized to the M2 phenotype. To confirm these data, we analyzed the expression of mannose receptor (CD206), another M2 marker, in the CD11b^+^ monocyte cell population in the ipsilateral ICH-injured brain using flow cytometry (Fig. 2g). The percentages of mannose receptor-expressing macrophages (CD206^+^/CD11b^+^/CD45^high^) increased to 68.5 % at 7 days post-ICH, whereas the CD206^+^ microglia population (CD206^+^/CD11b^+^/CD45^low^) increased to only 16.7 %. Taken together, these data suggest that the majority of macrophages in the ICH-injured brain are polarized to the M2 phenotype at delayed time points (3 and 7 dpi).

### Macrophage depletion attenuates recovery after ICH

**Fig. 3:**
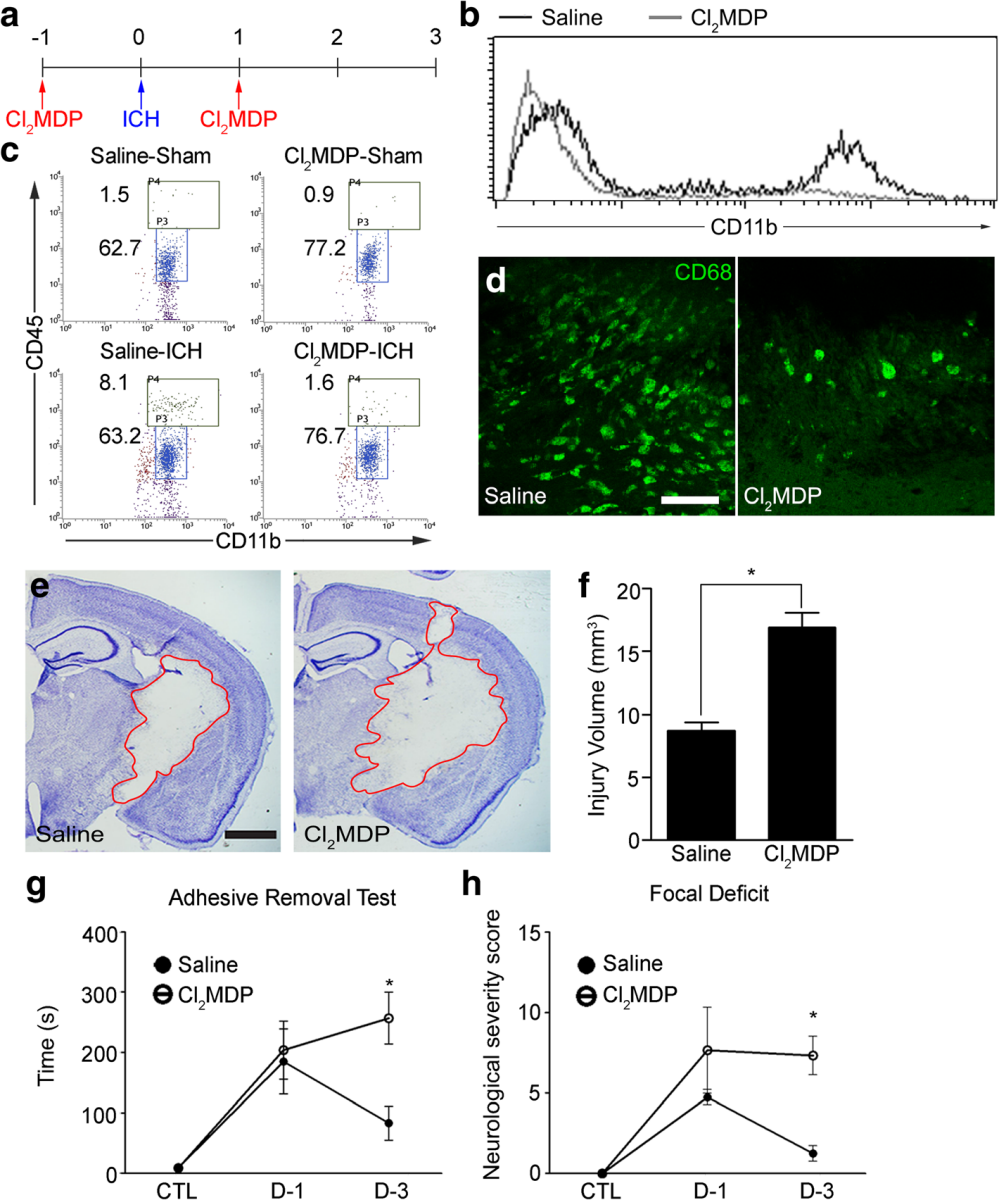
Depletion of peripheral monocytes in ICH injury. **a** Schematic diagram of the myeloid cell depletion protocols using Cl_2_MDP. **b** Blood cells obtained from saline (*black line*)- or Cl_2_MDP-treated ICH mice (*gray line*) were stained with anti-CD11b-FITC antibody and analyzed with flow cytometry. **c** Cell suspensions obtained from saline- or Cl_2_MDP-treated mouse brain were stained with anti-CD11b-FITC and anti-CD45-PE antibody and analyzed with flow cytometry. Representative flow cytometry dot plots are shown. **d** ICH injured brain sections were collected and stained with anti-CD68 antibody. Representative images of three independent experiments are shown (Scale bar, 50 μm). **e** One day after Cl_2_MDP injection, mice were subjected to ICH, and the brains were sectioned and stained with cresyl violet at 3 dpi. Representative pictures are shown (Scale bar, 1 mm). **f** Hemorrhagic injured volumes were quantified and presented in a graph (*n* = 4 per group, *p < 0.05). **g**, **h** After Cl_2_MDP or saline i.p. injection, mice were subjected to ICH, and neurological outcomes were evaluated by focal deficit and sticky tape removal time at 1 and 3 days after ICH (*n* = 5 for saline and Cl_2_MDP groups in ART; *n* = 4 for saline group, *n* = 3 for Cl_2_MDP group in focal deficit, *p < 0.05). Data are expressed as mean ± SEM

To assess the role of these M2 macrophages, we depleted the monocyte cell population by treating with clodronate liposome (Cl_2_MDP) and then subjected mice to ICH injury. Upon Cl_2_MDP injection (i.p.), 97 % of blood monocytes were depleted from the blood (Fig. 3b). In the ICH-injured brain, the percentage of CD11b^+^/CD45^high^ macrophages was increased to 8.1 % (saline-ICH) from 1.5 % (saline-sham) (Fig. 3c). However, in the Cl_2_MDP-treated mice brain, the macrophage population only increased to 1.6 % (Cl_2_MDP-ICH) from 0.9 % (Cl_2_MDP-sham). Meanwhile, the microglia (CD11b^+^/CD45^low^) populations were not significantly altered by Cl_2_MDP treatment (Fig. 3c). Decreased macrophage infiltration in the Cl_2_MDP-injected mouse brain was also confirmed by immunohistochemistry (Fig. 3d). The CD68^+^ macrophages in the ICH-injured brain were significantly reduced in Cl_2_MDP-treated mice (Fig. 3d). Cresyl violet staining of ipsilateral brain sections from Cl_2_MDP- or saline-treated mice showed that monocyte-depleted mice had larger infarct volumes after ICH compared to control mice (Fig. 3e and f). Consistent with the histological data, the Cl_2_MDP-treated mice displayed more severe motor function impairment as measured by the adhesive removal test (Fig. 3g) and focal deficits scoring (Fig. 3h) at 3 dpi. These findings indicate that brain-infiltrating M2 macrophages play a protective role by presumably facilitating recovery from ICH injury.

### Macrophages are polarized to M2 phenotype by soluble factors secreted by glial cells

**Fig. 4:**
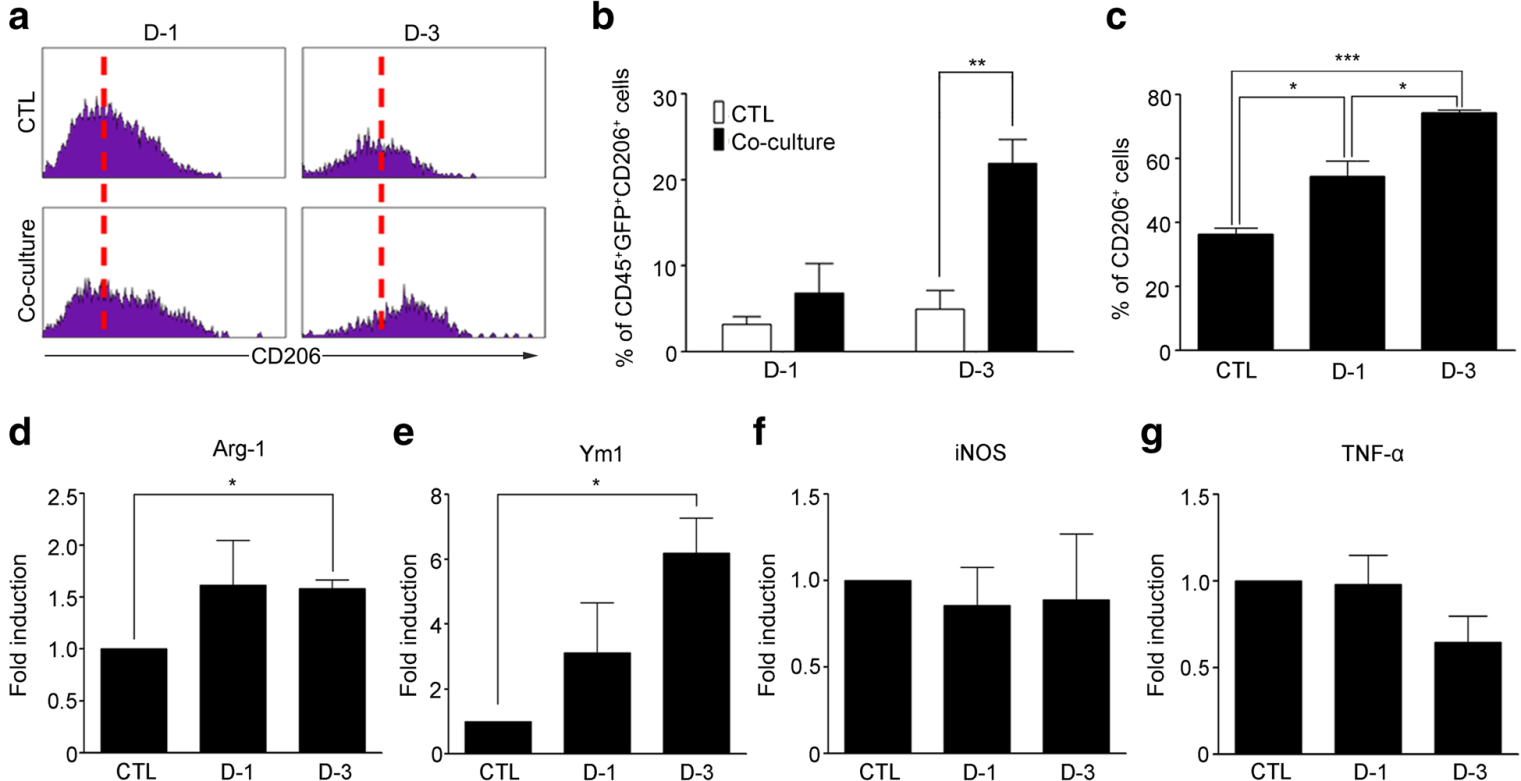
Soluble factors from mouse brain glial cells induce M2 polarization of BMDM. **a** BMDM from *Cx3cr1* ^*+/GFP*^ mice were cultured with or without primary glial cells from C57BL/6 mice in a 1:1 ratio for 1 or 3 days. Then the expression of CD206 (a M2 marker) was analyzed using flow cytometry. Representative histograms of CD206 expression in GFP^+^/CD45^+^ populations are shown. **b** The percentages of CD206^+^ populations in BMDM were calculated. Mean ± SEM of 3 independent experiments are shown (*p < 0.05). **c** BMDM were incubated in primary glia-conditioned media for 1 and 3 days. Then, the cells were harvested and stained with CD45-PE, CD11b-FITC, and CD206-APC for flow cytometry analysis. The percentages of CD206^+^ cells in CD45^+^/CD11b^+^ populations are analyzed. Mean ± SEM of 3 independent experiments are shown (*p < 0.05, ***p < 0.001). **d–g** Total RNA was isolated from BMDM at 1 or 3 days after glia-conditioned media treatment. Arg-1, Ym1, iNOS, and TNF-α mRNA expressions were measured using real-time RT-PCR. Mean ± SEM of at least 3 independent experiments are shown (*p < 0.05)

Since the macrophages in the injured brain were M2 phenotype, it is conceivable that brain microenvironment polarizes brain-infiltrating macrophages to the M2 phenotype. Supporting this possibility, a previous study has shown that bone marrow-derived macrophages (BMDM) exhibit an M2-like phenotype after co-culture with an astroglioma cell line [25]. To test if glial cells affect macrophage polarization toward the M2 phenotype, BMDM from *Cx3cr1*^*+/GFP*^ mice were co-cultured with primary cultured mouse brain glial cells, and mannose receptor expression was measured using flow cytometry. After co-culture with primary glia for 3 days, the percentage of CD206^+^ cells in the BMDM population (GFP^+^/CD45^+^) increased to 20 %, indicating polarization to the M2 phenotype (Fig. 4a and b). Notably, the percentage of CD206^+^ BMDM also increased to more than 60 % upon incubation in glia-conditioned media for 3 days (Fig. 4c), indicating that direct cell contact between BMDM and glia is not required for M2 polarization. In addition, the expression of other M2 genes Arginase-1 and Ym1 increased by 1.5- and 6-fold, respectively (Fig. 4d and e). However, expression of iNOS and TNF-α mRNA, M1 marker genes, was not significantly upregulated after glia-conditioned media treatment (Fig. 4f and g). These data suggest that glia-derived soluble factor(s) induce M2 polarization of the BMDM, which might account for the M2 polarization of macrophages observed in the ICH-injured brain.

## Discussion

Although the function of brain-infiltrating immune cells in ischemic stroke has been well documented, their role in hemorrhagic stroke is not completely understood. To our knowledge, this study is the first to assert that macrophages infiltrating brain parenchyma after ICH might play a beneficial role in recovery from ICH injury. Our data are in contrast to the recent report by Hammond et al., which showed that brain-recruited inflammatory monocytes exacerbate disability following ICH [26]. In this study, they showed that immune cells recruited to the injured brain after ICH are mainly CCR2^+^/Ly6C^+^ inflammatory macrophages that peaked at 1–3 dpi. Such discrepancy might simply be due to differences in their injury model, since they characterized the phenotype of macrophages in an ICH model using autologous blood infusion. They also showed that these inflammatory macrophages contribute to acute neurological deficit by depleting inflammatory macrophages using CCR2 antibody. CCR2 is expressed on inflammatory macrophages but not on CX3CR1^+^ monocytes [27]. Thus, the treatment of this antibody might have depleted only inflammatory monocytes but not CCR2-negtive M2 macrophages. Therefore, it is possible that the contribution of anti-inflammatory or wound-healing M2 macrophages that are recruited after 3 dpi might have been overlooked in their study.

It should also be noted that clodronate liposome may deplete both monocytes and other myeloid cells, such as dendritic cells. A recent study showed that dendritic cells were also recruited to the injured brain after ICH [28]. Thus, we cannot rule out the possibility that the effects of clodronate liposome on ICH injury could be due to both monocyte and dendritic cell depletion. In the present study, we did not analyze the relative contributions of brain-infiltrating macrophages and dendritic cells, which warrants future investigation.

Our data clearly show that brain-infiltrating macrophages play distinct roles depending on the stroke model used. In a murine ischemic stroke model, macrophages played a detrimental role by exacerbating neuronal cell death [17]. In this model, macrophages in the peri-infarct brain region were gradually transformed into M1 phenotype, which contributed to the detrimental effects [17]. In collagenase-induced ICH, M2 phenotype macrophages expressing Arginase-1, Ym1 and CD206 were obtained at delayed time points (3 and 7 dpi). Thus far, it is not clear why macrophages are activated differently depending on the stroke model. Although purely speculative, it is presumed that the microenvironment of the ischemic-injured brain might be distinct from that of the hemorrhagic brain. In this regard, it is of note that ischemic neurons are able to prime microglia polarization to M1 phenotype [17].

In this study, we did not elucidate how M2 macrophages exert their neuroprotective effects in ICH. It is well-known that M2 polarized macrophages contribute to wound-healing by producing various wound-repair genes [29]. It has been reported that brain-infiltrating macrophages contribute to damaged neurovascular unit repair through TGF-β1 secretion [30]. Therefore, it is possible that brain-infiltrating M2 macrophages reduce infarct damage by contributing to the wound-healing process in the injured brain tissue. In addition, studies have shown that M2 polarized microglia protect neurons by secreting neurotrophic factors such as BDNF or IGF [31, 32]. In this regard, it is also conceivable that M2 macrophages protect neurons from ICH injury by producing such neurotrophic factors.

In our effort to elucidate mechanisms of macrophage M2 polarization in the ICH brain, we found that glia-derived soluble factors have a tendency to drive M2 polarization of BMDM. After infiltration into inflamed tissue, the phenotype and activation of macrophages can be dynamically changed by the microenvironment of the recruiting tissue [33, 34]. In ICH-injured brains, brain-infiltrating macrophages most likely encounter a brain microenvironment consisting of glial cells in the peri-hematoma region [35, 36]. Because a co-culture system is widely accepted to study the influence of tissue microenvironment on macrophages, we postulated that a mixed glia culture in vitro might recapitulate the brain microenvironment of brain-infiltrating macrophages during ICH, and tested macrophage polarization in co-culture with mixed glia. Indeed, our data showed that primary glial cell-conditioned media can induce the macrophage M2 phenotype. These data are in line with a previous document showing that astrocytes can induce M2 activation of BMDM via the upregulation of miR-124 [25]. In addition to the glia-derived factors, various neuron-derived factors are implicated in driving the macrophage M2 phenotype, including CX3CL1 and fragmented myelin [37, 38]. In this regards, we do not exclude the possibility that macrophages are polarized to M2 phenotype due to neuron-derived molecules in the ICH-injured brain. Although we have not clearly demonstrated the mechanism, our data suggest that brain-infiltrating macrophages are polarized to the M2 phenotype due to the microenvironment of the ICH-injured brain, which facilitates recovery from ICH injury.

## Conclusions

In this study, we found that alternatively activated (M2) macrophages accumulate in the ICH-injured brain. These macrophages contribute to recovery from ICH at a delayed time. Additionally, we showed that soluble factors released from glial cells drive macrophages to the M2 phenotype. Our data suggest that brain-infiltrating macrophages are polarized to the M2 phenotype and facilitate recovery after ICH.

## Abbreviations

ART: adhesive removal test
BBB: blood brain barrier
BMDM: bone marrow-derived macrophages
CD206: mannose receptor
Cl_2_MDP: clodronate liposome
DMEM: Dulbecco’s Modified Eagle’s medium
dpi: day post-injury
ICH: intracerebral hemorrhage
IL: interleukin
LPS: lipopolysaccharide
M1: classically activated macrophage
M2: alternatively activated macrophage
NEAA: non-essential amino acids
rhM-CSF: recombinant human macrophage colony stimulating factor
RT: room temperature
TLR2: toll-like receptor 2
